# Late-onset argininosuccinic aciduria associated with hyperammonemia triggered by influenza infection in an adolescent: A case report

**DOI:** 10.1016/j.ymgmr.2020.100605

**Published:** 2020-05-15

**Authors:** Yoshimitsu Osawa, Aya Wada, Yoshiaki Ohtsu, Kenji Yamada, Takumi Takizawa

**Affiliations:** a Department of Pediatrics, Gunma University Graduate School of Medicine, Japan; b Department of Pediatrics, Shimane University Faculty of Medicine, Japan

**Keywords:** Argininosuccinic aciduria, Argininosuccinic acid lyase deficiency, Late-onset, Hyperammonemia, Influenza A, Adolescence, ASA, argininosuccinic aciduria, ASL, argininosuccinate lyase

## Abstract

Hyperammonemia is a typical symptom of urea cycle disorders. While early-onset argininosuccinic aciduria (ASA) can often be detected by hyperammonemia, patients with late-onset ASA predominantly present with psychomotor retardation and mental disorders. However, in late-onset ASA that develops during early childhood, hyperammonemia can sometimes be caused by acute infections, stress, and reduced dietary intake. Here, we report the case of a 14-year-old boy with late-onset ASA associated with hyperammonemia that was triggered by an influenza A infection. Due to the infection, he presented with a fever and was unable to eat food or take oral medication. He then experienced restlessness, a disturbance in his level of consciousness, and seizures. Hyperammonemia (3286 μg/dL, reference value ≤100 μg/dL) was detected. He was biochemically diagnosed with ASA based on increased serum and urinary argininosuccinic acid levels. Additionally, genetic testing revealed compound heterozygous mutations in the *ASL* gene: c.91G > A(p.Asp31Asn) and c.1251-1G > C. This case revealed that in late-onset ASA, hyperammonemia can occur not only in early childhood but also during adolescence. Late-onset ASA may have a very broad clinical spectrum that includes hyperammonemia. We suggest that urea cycle disorders such as ASA must be considered when patients present with hyperammonemic decompensation during adolescence.

## 1 Introduction

Argininosuccinic aciduria (ASA; OMIM 207900) is an autosomal recessive congenital dysfunction in the urea cycle that was first reported in 1958 [1]. ASA is caused by an argininosuccinate lyase (ASL; EC 4.3.2.1; MIM# 608310) deficiency. ASL catalyzes the fourth step in the urea cycle, in which argininosuccinic acid is cleaved to produce arginine and fumarate. *ASL* (NM_000048.3) is the only gene for which mutations are known to be associated with ASA. *ASL* is located on chromosome 7, locus 7q11.21 [2]. ASA is the second or third common urea cycle disease, with an estimated overall incidence of one in 220,000 newborns in North America and Europe [3,4]. In Japan, a nationwide expanded newborn screening that included urea cycle disorders has been carried out only since 2014.

The age of onset for ASA ranges from the neonatal period to adulthood, but most patients develop the condition by early childhood. ASA is classified into two forms: early-onset and late-onset. Patients with the early-onset form present with life-threatening hyperammonemic crises, seizures, hypothermia, hyperventilation, vomiting, and lethargy within the neonatal period. Patients with the late-onset form range from episodic hyperammonemia after the neonatal period to psychomotor retardation and mental disorders in the absence of recurrent hyperammonemia episodes [[5], [6], [7]]. Episodic hyperammonemia can be triggered by acute infection, stress, reduced dietary intake, and/or medication during the neonatal-to-early-infancy period. Trichorrhexis nodosa and a dislike of protein-rich food are sometimes exhibited. The main biochemical finding is the accumulation of ASA in the urine and blood. Additionally, plasma amino acid analysis typically reveals elevated citrulline levels. Molecular genetic testing of *ASL* is useful for diagnosis when the biochemical findings are equivocal. Some types of *ASL* mutations are suggested to have genotype phenotype correlations [[8], [9], [10]].

Dietary restriction of protein, arginine supplementation, and alternative pathway therapy (sodium benzoate and sodium phenyl butylate) are the mainstay treatments in the long term management of ASA [11].

In previous reports, late-onset ASA was usually diagnosed in childhood and was rarely associated with hyperammonemia [4,12,13]. Therefore, we assumed that patients with ASA who exhibited hyperammonemia as the trigger of diagnosis in adolescence were very rare. Here, we report the case of a 14-year-old boy with late-onset ASA who presented with episodic hyperammonemia triggered by an influenza type A infection.

## 2 Case report

Our patient was a 14-year-old boy with an uneventful neonatal period. He was not tested by expanded newborn screening covering urea cycle disorders because it had not yet been initiated in Japan. His psychomotor development was gradually delayed after 1 year of age. Because brain magnetic resonance imaging (MRI) and electroencephalography revealed no abnormality, the cause of psychomotor retardation was not identified at that time. He had attended a special-needs class for handicapped children since entering elementary school. Until school age, he had sometimes vomited when he was not feeling well and when his environment changed. It was unknown whether he had previously developed hyperammonemia because he had not been measured for ammonia levels until he was 14 years old.

When he was 14 years old, he was capable of coping with daily living and could converse. He could perform simple multiplication and division, but it was difficult for him to understand the content of problems from the text. He was one of the slowest runners among his classmates. His hair was mainly black but partially brown and damaged-looking. He subconsciously avoided protein-rich foods such as meat, fish, and milk; he preferred rice and vegetables. There was no family history of a sudden unexpected death, psychomotor retardation, or congenital metabolic disorders. There was no consanguineous marriage. He was the second child and his elder brother was healthy.

At 14 years of age, he presented with a fever and was unable to eat enough one day before admission. He then experienced restlessness and disturbed consciousness. He was admitted at a local hospital. Influenza type A was detected by rapid influenza antigen tests. Treatment with midazolam was initiated due to general convulsions. Because hyperammonemia (515 μg/dL, reference value ≤100 μg/dL) was detected at admission, and his ammonia levels were elevated 2 h after admission (849 μg/dL), he was transferred to our intensive care unit.

**Fig. 1:**
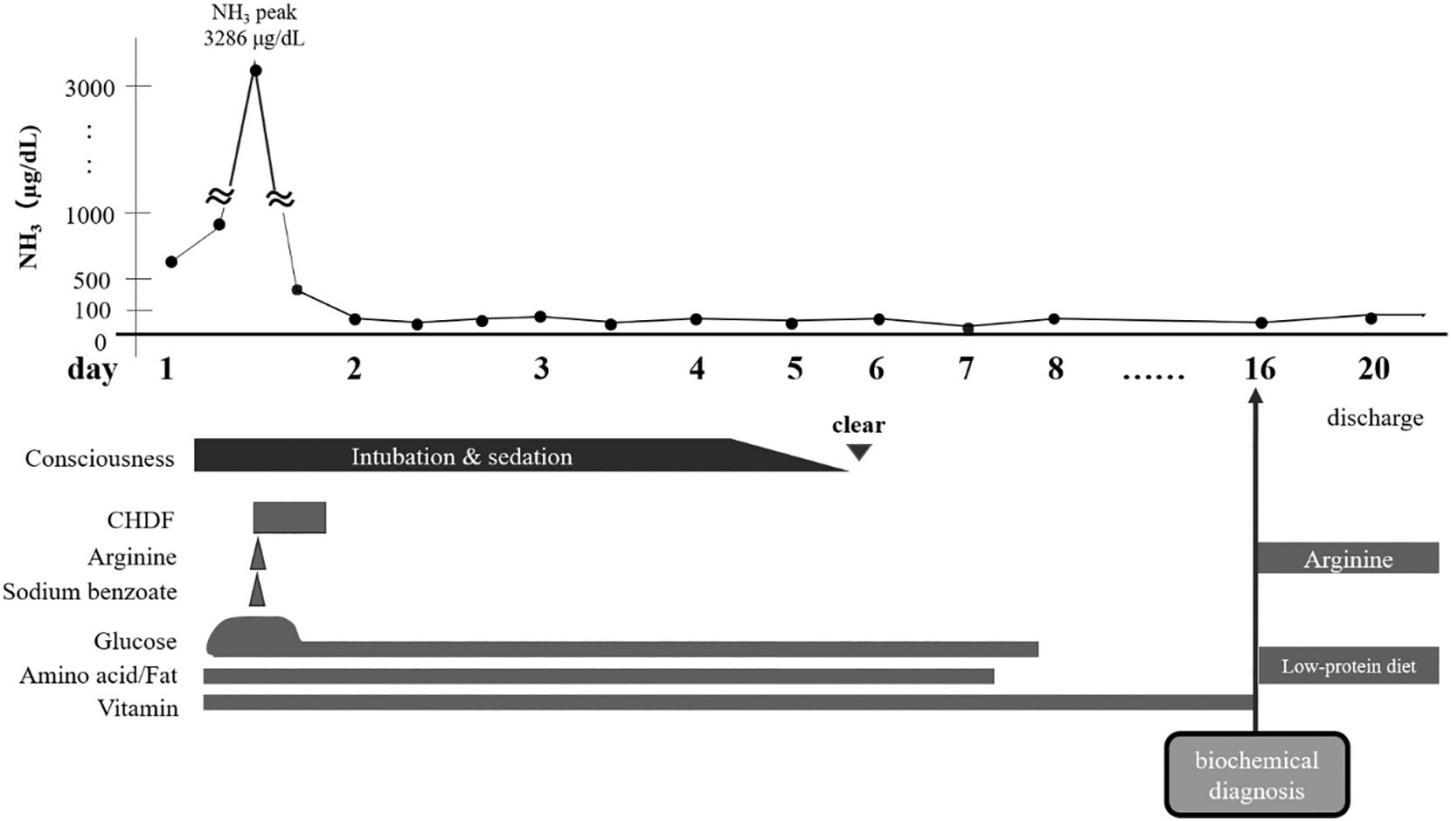
Clinical course during the acute phase. CHDF: continuous hemodiafiltration.

Arginine and sodium benzoate were administered to treat the hyperammonemia. In addition, because the ammonia levels were 3286 μg/dL on admission to our hospital, continuous hemodiafiltration was initiated; thereafter, the hyperammonemia promptly improved. He received peramivir as a treatment for influenza. Head computed tomography revealed brain edema; methylprednisolone and mannitol were used as a treatment for encephalopathy. On hospital day 4, midazolam was stopped, and his consciousness disorder gradually improved (Fig. 1).

His plasma citrulline levels were mildly elevated (105.7 nmol/mL, reference value 17.9–48.0 nmol/mL), and his plasma arginine and ornithine levels were low (8.3 nmol/mL, reference value 42.6–141.2). Urinary excretion of orotic acid and uracil were detected by urinary organic acid analysis. Serum and urinary argininosuccinic acid levels were highly elevated (serum: 431.03 μmol/L, reference value <1.5 μmol/L; urine: 24577 μmol/mmol creatinine), and he was biochemically diagnosed with ASA (Table 1). Treatment consisting of a low-protein diet (≤1.5 g/kg per day) and oral arginine tablets (5 g per day) was initiated. He was discharged on the 20th day after admission. After discharge, he was followed-up regularly as an outpatient and was administered sodium phenylbutyrate in addition to the treatments above. There was no change in the baseline neurological status compared to that before decompensation. Hyperammonemia was not detected until he was 17 years of age, when this report was written.

**Table 1:** Results of biochemical examinations on admission to our hospital.

**【Arterial blood test】**								
WBC	4500	/μL	AST	105	U/L	NH_3_	3286	μg/dL
Neu	84.6	%	ALT	85	U/L	Glucose	325	mg/dL
Lym	8.9	%	LDH	374	U/L	Lactate	26	mg/dL
Eos	0	%	ALP	839	U/L	Pyruvate	1	mg/dL
Bas	0	%	γGTP	18	mg/dL			
Mon	6.5	%	BUN	7	mg/dL	**〔Blood gas analysis (intubation)〕**		
Hb	13.6	g/dL	Cre	0.46	mEq/L	pH	7.464	
Plt	23.8	×10^3^/μL	Na	139	mEq/L	pCO_2_	22.6	mmHg
			K	4.8	mEq/L	pO_2_	222	mmHg
PT	85	%	Cl	106	mEq/L	HCO_3_^−^	16.2	mEq/L
APTT	69	Sec	CRP	0.09	mg/dL	BE	−6.8	mEq/L
Fibrinogen	189	mg/dL						
D-dimer	4.3	μg/mL						
**【Blood amino acid analysis】**								
	Cit	105.7	nmol/mL	(17.9–48.0 nmol/mL)				
	Orn	8.3	nmol/mL	(42.6–141.2 nmol/mL)				
	Arg	25	nmol/mL	(31.8–149.5 nmol/mL)				
	Asp	34.2	nmol/mL	(trace–7.2 nmol/mL)				
	Asn	17.6	nmol/mL	(43.8–90.6 nmol/mL)				
	Glu	207.2	nmol/mL	(12.2–82.7 nmol/mL)				
	Gln	893.6	nmol/mL	(418–739.8 nmol/mL)				
	Ala	424.1	nmol/mL	(258.8–615.2 nmol/mL)				
**【Urine organic acid analysis】**								
Urinary excretion of lactate, pyruvate, acetoacetic acid, 3-hydroxybutyric acid, α-keto acid, uracil, and orotic acid was increased.								
Serum argininosuccinic acid			431.03	μmol/L (<1.5 μmol/L)				
Urine argininosuccinic acid			24577	μmol/mmol Cre				

Ala: alanine, ALP: alkaline phosphatase, ALT: alanine aminotransferase, Arg: arginine, Asn: asparagine, Asp: aspartic acid, AST: aspartate aminotransferase, bas: basophil, BE: base excess, BUN: blood urea nitrogen, Cit: citrulline, Cre: creatinine, CRP: C-reactive protein, eos: eosinophil, γGTP: γ-glutamyl transpeptidase, Gln: glutamine, Glu: glutamic acid, LDH: lactate dehydrogenase, lym: lymphocyte, mon: monocyte, neu: neutrophil, Orn: ornithine.

He underwent brain MRI twice during hospitalization, on the 4th and 14th hospital days. The first MRI scan showed high signal intensity at the bilateral thalamus and part of the left frontal deep white matter. However, the second MRI scan showed a tendency toward recovery of these findings. We could not estimate his cognitive function after the hyperammonemia crisis.

Subsequently, using the Japanese genetic panel for inherited metabolic disorders with next-generation sequencing, compound heterozygous mutations in the *ASL* gene were detected including a c.91G > A (p.Asp31Asn) missense mutation and a c.1251-1G > C splice site mutation.

## 3 Discussion

This is a rare report of a patient with *de novo* ASA who presented with hyperammonemia at 14 years of age. We assumed that patients with ASA who exhibited hyperammonemia as the trigger of diagnosis in adolescence were very rare. Because ASA has a very broad clinical spectrum, particularly in late-onset cases, psychiatric symptoms without hyperammonemia often lead to the diagnosis of late-onset ASA [5]. In addition, the onset time differs slightly depending on the geographical region. For example, North American and European patient cohorts indicated that the median age of diagnosis was 365 days and 210 days, respectively; the third quartile date was 700 days and 1080 days in 219 North American and 18 EU patients, respectively [4]. Therefore, most ASA patients were diagnosed by the age of 3 years. In our case, a definite diagnosis could not be reached although the patient had exhibited mild mental retardation from the age of 1 year and vomiting that may be due to transient hyperammonemia; he did not receive ASA management, which resulted in critical hyperammonemia at 14 years of age. Additionally, the incidence of ASA is much lower in Asia than in Europe or North America [14]. This may potentially explain why ASA was not considered when he was examined during early childhood.

It is worth noting that acute hyperammonemia can present in late-onset ASA. In Spain, it was previously reported that only 1 out of 6 late-onset ASA patients had hyperammonemia and encephalopathy [12]. In Malaysia, 1 of 3 late-onset patients with ASA was diagnosed at 14 months of age, triggered by feeding lethargy and coma [13]. In late-onset ASA, patients with acute symptoms such as hyperammonemia are fewer in number than those with neuropsychiatric symptoms at diagnosis [12]. In addition, a UK study showed that 50% of late-onset ASA patients had normal ammonia levels at diagnosis [15]. The patients with acute symptoms whose onset age is known are diagnosed with late-onset ASA by early childhood [13]. However, ASA is sometimes diagnosed only in middle or old age [16,17]. There are reports of siblings who were diagnosed with ASA triggered by psychiatric symptoms in their 40s and 60s. From these reports, we assumed that, after adolescence, neuropsychiatric symptoms were the key factor in the diagnosis of late-onset ASA. However, from our case, we suggest that it should be taken into consideration that patients with late-onset ASA can develop not only neuropsychiatric symptoms but also acute symptoms triggered by hyperammonemic encephalopathy after adolescence.

In this case, hyperammonemia was not detected after decompensation, and we supposed that the patient had normal ammonia levels when in stable condition, despite having psychiatric symptoms. In the current literature, ASA patients performed worse in cognitive testing, compared to other urea cycle disorders, despite a lower frequency of hyperammonemic events; this suggests that other ammonia-independent pathomechanisms, such as disturbed NO metabolism causing cerebral oxidative/nitrosative stress, might underlie the disease [18]. Abnormal catecholamine levels caused by downregulation of tyrosine hydroxylase due to defective nitrosylation results in an abnormal response to stress and in increased seizure sensitivity in mice [19]. This ammonia-independent pathomechanism may be related to the broad phenotypic spectrum of late-onset ASA. In our case, the mild psychiatric symptoms and development of hyperammonemic encephalopathy in adolescence may be related to this ammonia-independent pathomechanism.

In our case, compound heterozygous mutations in the *ASA* gene (c.91G > A (p.Asp31Asn) and c.1251-1G > C) were detected. These mutations were found to be pathogenic *in silico* according to Mutation taster (http://www.mutationtaster.org/) and Polyphen2 (http://genetics.bwh.harvard.edu/pph2), and are, therefore, considered to be the cause of illness in the current case. The p.Asp31Asn mutation has been detected in patients with the late-onset phenotype [9]. The c.1251-1G > C mutation has not been previously reported. Some types of *ASL* mutations are suggested to have genotype-phenotype correlations [[8], [9], [10]]. These mutation might be relevant to the development of hyperammonemia after the neonatal period. Correlations between residual ASL activity and certain clinical endpoints (*e.g.*, plasma NH_4_^+^ concentration and number of hyperammonemic events per years) are suggested [10]. However, because we had not previously measured residual ASL activity, we could not accurately predict the disease severity. Predicting the severity of ASA is difficult when novel mutations are detected as their effects are unknown.

## 4 Conclusion

This rare case report describes a 14-year-old boy with late-onset ASA and hyperammonemia triggered by an influenza infection. We suggest that late-onset ASA can manifest as not only chronic psychiatric symptoms but also as acute hyperammonemia crises, even in adolescents. ASA should be considered as a differential diagnosis when patients present with hyperammonemia, regardless of their age, and with neurobehavioral abnormalities at a younger age; these are key symptoms of late-onset ASA.

## Contributions of individual authors

Y. Osawa was the attending physician of the patient and wrote the initial draft of the manuscript. A. Wada was the attending physician of the patient in the outpatient facility and obtained information from the patient. Y. Ohtsu and K. Yamada advised the design of the initial draft of the manuscript and revised it. T. Takizawa critically revised this draft for important intellectual content and provided the final approval to submit this article.

## Declarations of Competing Interest

The authors have no conflicts of interest to declare.

## Details of funding

This research was supported by Grant-in-Aid Scientific Research from the 10.13039/501100001691Japan Society for the Promotion of Science (Grant Number JP 19K17975).

## Patient consent

Written informed consent was obtained from the patient for publishing this report.
